# Serum Cystatin C as a Potential Predictor of the Severity of Multiple System Atrophy With Predominant Cerebellar Ataxia: A Case-Control Study in Chinese Population

**DOI:** 10.3389/fnins.2021.663980

**Published:** 2021-09-10

**Authors:** Fei Ye, Tianzhu Wang, Huan Li, Jie Liang, Xiaoxin Wu, Wenli Sheng

**Affiliations:** ^1^Department of Neurology, The First Affiliated Hospital, Sun Yat-sen University, Guangzhou, China; ^2^Guangdong Provincial Key Laboratory of Diagnosis and Treatment of Major Neurological Diseases, The First Affiliated Hospital, Sun Yat-sen University, Guangzhou, China; ^3^Department of Neurology, The First Affiliated Hospital of Chongqing Medical University, Chongqing, China

**Keywords:** cystatin C, multiple system atrophy, unified multiple system atrophy rating scale, disease severity, case-control study

## Abstract

**Objective:** Multiple system atrophy (MSA) is a serious neurodegenerative disease that is charactered by progressive neurological disability. The aim of this study was to investigate the correlation of serum oxidant factors with the severity of MSA.

**Methods:** A total of 52 MSA patients and 52 age- and gender- matched healthy subjects were retrospectively enrolled in this study. Enzymatic colorimetric methods were used to assay the concentrations of uric acid (UA), serum creatinine (Scr), blood urea nitrogen (BUN), and cystatin C (Cys-C). Disease severity was evaluated by the Unified Multiple System Atrophy Rating Scale (UMSARS). The disease progression rate was defined by the change in UMSARS-IV (global disability score, GDS) over a 1-year period.

**Results:** Comparisons between the two groups revealed that there were no significant differences in terms of serum Scr (70.81 ± 13.88 vs. 70.92 ± 14.19 μmol/L, *p* = 0.967). However, the serum levels of the other three biomarkers were significantly higher in the MSA patients (UA: 325.31 ± 84.92 vs. 291.19 ± 64.14 μmol/L, *p* = 0.023; BUN: 5.68 ± 1.67 vs. 4.60 ± 1.24 mmol/L, *p* < 0.001; Cys-C: 0.96 ± 0.15 vs. 0.89 ± 0.14 mg/L, *p* = 0.024). In addition, Pearson correlation analyses revealed that only serum Cys-C was significantly correlated to GDS (*r* = 0.281, *p* = 0.044). Subgroup analysis further demonstrated that serum Cys-C was the only factor that was positively associated with the disease severity in patients with MSA and predominant cerebellar ataxia (MSA-C) (*r* = 0.444, *p* = 0.018); there was no significant association in MSA patients with predominant Parkinsonism (MSA-P) (*r* = 0.118, *p* = 0.582). MSA-C patients with severe disability were shown to express higher serum levels of Cys-C than patients with mild disability (1.03 ± 0.13 vs. 0.88 ± 0.12 mg/L, *p* = 0.009). Finally, Kaplan-Meier plots revealed a significant difference in the 5-year probability of survival from severe disability between MSA-C patients with high- and low-concentrations of serum Cys-C (Log-rank test: X^2^ = 4.154, *p* = 0.042). ROC curve analysis confirmed that serum Cys-C exhibits good performance as a biomarker (AUC = 0.847).

**Conclusion:** Our research indicated that oxidative stress plays a vital role in MSA. Serum Cys-C represents a potential prognostic biomarker to evaluate the severity of disease in patients with MSA-C.

## Introduction

Multiple system atrophy (MSA) is an adult-onset, sporadic, and rapidly progressive neurodegenerative disease that is characterized by autonomic failure in connection with Parkinsonism and/or cerebellar ataxia (Stefanova et al., 2009). The generation of α-synuclein usually acts as a pathological hallmark of MSA and other Parkinson-related diseases and involves the accumulation of argyrophilic filamentous glial cytoplasmic inclusions from the cerebrospinal fluid (Stefanova et al., 2009). The misfolding of α-synuclein is increasingly being considered as a key initial event in the pathophysiological cascade of MSA. Recent studies have successfully described the epidemiological and clinical characteristics of this disease, although the specific causes and mechanisms involved still remain unclear. The rapid progression of this disease, and the lack of an appropriate therapeutic intervention, results in a serious deterioration of neurological function and a fatal outcome.

A significant body of evidence now indicates that oxidative stress plays a key role in triggering and/or exacerbating MSA (Nee et al., 1991; Vanacore et al., 2005; Fernagut and Tison, 2012; Stefanova et al., 2012; Zhou et al., 2016). Oxidative stress can contribute to neuronal damage and can modulate intracellular signaling pathways, ultimately leading to neuronal death by apoptosis or necrosis (Calabrese et al., 2010). Research has shown that several serum oxidant factors may act as important biomarkers to predict the disease severity in neurodegenerative diseases, such as Parkinson’s disease (PD) (Hu et al., 2016; Sampat et al., 2016) and amyotrophic lateral sclerosis (ALS) (Tetsuka et al., 2013; Paganoni et al., 2018; van Eijk et al., 2018), including uric acid (UA), serum creatinine (Scr), and cystatin C (Cys-C). However, its controversy remains as to whether UA and Scr could act as predictive factors for MSA (Lee et al., 2011; Cao et al., 2013, 2016; Chen et al., 2015; Sakuta et al., 2016; Fukae et al., 2017). Furthermore, the roles of Cys-C and blood urea nitrogen (BUN) have yet to be investigated in patients with MSA. In this case-control study, we aimed to evaluate differences in these biomarkers between MSA patients and healthy subjects, and to evaluate the potential correlations between these biomarkers and the severity of MSA in patients from a Chinese population.

## Materials and Methods

### Study Subjects

**FIGURE 1:**
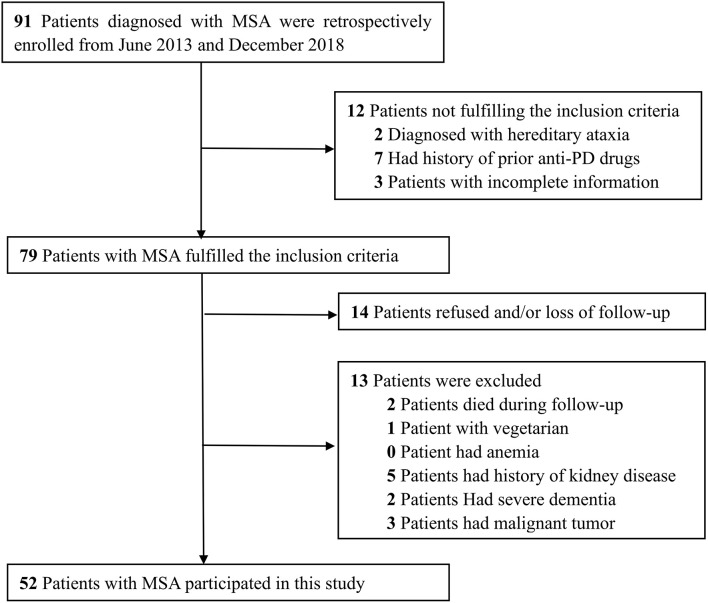
Flow chart presenting the process of patient inclusion and exclusion of this study.

This study was a case-control study that took place between June 2013 and December 2018 and involved Chinese patients with MSA. A total of 91 Chinese patients previously diagnosed with MSA were retrospectively identified via diagnosis-specific code (ICD-10 code G90.301). Patients were eligible for inclusion in this study if they met the following criteria: (1) if diagnosis had been made on the basis of the second consensus criteria (Gilman et al., 2008), (2) there was no history of a previous diagnosis of PD and medicated with anti-PD drugs, (3) a complete set of clinical data was available. We excluded patients who were vegetarian, those with anemia and/or kidney disease, those with severe dementia, and those with advanced stages of cancer. After applying these criteria, we identified 52 MSA patients for inclusion (Figure 1). In addition, 52 age- and gender-matched subjects were selected from the database held by our Health Management Center as controls. These controls were neurologically normal and free of a family history of movement disorders. All participants provided written informed consent. The study was approved by the Ethics Committees of the First Affiliated Hospital of Chongqing Medical University and the First Affiliated Hospital of Sun Yat-sen University.

### Sample Preparation and Methods

Serum levels of UA, Scr, BUN, and Cys-C were determined at baseline by an enzymatic colorimetric method and an automatic analyzer (Type 7600; Hitachi Ltd., Japan) in our clinical laboratory. We also recorded a range of clinical information, including gender, age, disease duration (defined as the period between symptom onset and the last hospitalization), history of hypertension, diabetes mellitus, cigarette use, and alcohol abuse. The disease severity of MSA was evaluated by the Unified Multiple System Atrophy Rating Scale (UMSARS) (Wenning et al., 2004). The disease progression rate was defined as the change in UMSARS-IV (global disability score, GDS) over a period of 1 year (Fukae et al., 2017).

### Statistical Analysis

Continuous variables are presented as mean ± standard deviation for age, disease duration, UMSARS, disease progression rate, and the concentrations of UA, Scr, BUN, and Cys-C. Categorical variables are presented as percentages, including gender, history of hypertension, diabetes mellitus, cigarette use, and alcohol abuse. The Chi-squared test was used to compare categorical variables between MSA patients and healthy subjects while the *t*-test was used to identify differences in continuous variables. The Pearson correlation coefficient was used to determine the associations between serum oxidant factors and disease severity and to identify significant biomarkers in subgroup analysis. The subgroups included MSA with predominant Parkinsonism (MSA-P) and MSA with predominant cerebellar ataxia (MSA-C) (Gilman et al., 2008). The Kaplan-Meier method was used to explore the probability of survival from severe disability. Receiver operating characteristic (ROC) curves were applied to evaluate the performance of significant biomarkers. All statistical analyses were performed using the Statistical Program for Social Science (SPSS, version 26) and GraphPad Prism (version 9). The significance level was set to 0.05 for all tests.

## Results

### Demographic Characteristics

Demographic characteristics and clinical data related to the MSA patients and healthy subjects are listed in Table 1. A total of 44 patients diagnosed with probable MSA and 8 of them diagnosed with possible MSA. The mean age of the MSA patients was 57.8 ± 9.1 years; over 65% of the subjects were male. The mean UMSARS-I, -II, -IV and total scores were 9.6 ± 4.3, 13.6 ± 5.1, 2.2 ± 1.1, and 25.4 ± 8.7, respectively. For these patients, the mean disease duration was 2.3 ± 1.1 years and the calculated disease progression rate was 1.4 ± 1.2. There were no significant differences between the groups in terms of age, gender distribution, or history of vascular risk factors. We also divided the patients into MSA-C (*n* = 28) and MSA-P (*n* = 24) subgroups; there were no significant differences between these groups with regards to the clinical parameters observed in this study (Table 2).

**TABLE 1:** The clinical characteristics of MSA patients and healthy subjects.

	MSA patients (*n* = 52)	Healthy subjects (*n* = 52)	*p*
Age (years)	57.8 ± 9.1	57.7 ± 9.1	0.974
Male	35 (67.3%)	35 (67.3%)	1.000
Risk factors			
Hypertension	1 (1.9%)	2 (3.8%)	0.558
Diabetes mellitus	4 (7.7%)	4 (7.7%)	1.000
Smoking	17 (32.7%)	19 (36.5%)	0.680
Drinking	13 (25.0%)	14 (26.9%)	0.823
Diagnosis			
Probable MSA	44 (84.6%)		
Possible MSA	8 (51.4%)		
Disease duration	2.7 ± 2.8		
UMSARS			
UMSARS-I	9.6 ± 4.3		
UMSARS-II	13.6 ± 5.1		
UMSARS-IV	2.2 ± 1.1		
Total score	25.4 ± 8.7		
Progression rate	1.4 ± 1.2		

**TABLE 2:** A comparison of clinical parameters between MSA-C and MSA-P subgroups.

	MSA-C (*n* = 28)	MSA-P (*n* = 24)	*p*
Age (year*s*)	58.0 ± 9.4	57.5 ± 9.0	0.833
Male	16 (57.1%)	19 (79.2%)	0.091
Risk factors			
Hypertension	0 (0.0%)	1 (4.2%)	0.275
Diabetes mellitus	2 (7.1%)	2 (8.3%)	0.872
Smoking	9 (32.1%)	8 (33.3%)	0.927
Drinking	9 (32.1%)	4 (16.7%)	0.199
Diagnosis			
Probable MSA	25 (89.3%)	19 (79.2%)	0.313
Possible MSA	3 (10.7%)	5 (20.8%)	
Disease duration	2.7 ± 2.1	2.8 ± 3.5	0.838
UMSARS			
UMSARS-I	8.9 ± 3.7	10.5 ± 4.8	0.172
UMSARS-II	14.5 ± 5.3	12.5 ± 4.6	0.164
UMSARS-IV	2.2 ± 1.1	2.3 ± 1.1	0.710
Total score	25.5 ± 8.3	25.3 ± 9.4	0.921
Progression rate	1.2 ± 1.2	1.7 ± 1.2	0.301

### Serum Oxidative Biomarkers in MSA Patients

**FIGURE 2:**
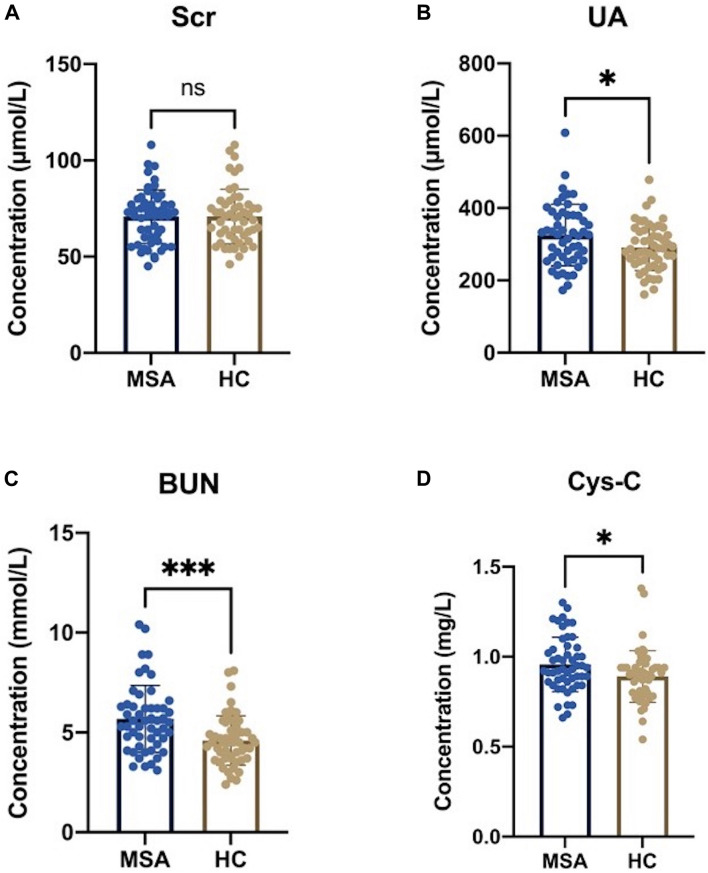
Serum oxidative biomarkers in MSA patients and healthy subjects. **(A)** There was no statistical significance in serum Scr levels between MSA patients and healthy controls (HCs) (70.81 ± 13.88 vs. 70.92 ± 14.19 μmol/L, *p* = 0.967). **(B)** The serum levels of UA were significantly higher in MSA patients than in HCs (325.31 ± 84.92 vs. 291.19 ± 64.14 μmol/L, *p* = 0.023). **(C)** The serum levels of BUN were significantly higher in MSA patients than in HCs (5.68 ± 1.67 vs. 4.60 ± 1.24 mmol/L, *p* < 0.001). **(D)** The serum levels of Cys-C was significantly higher in MSA patients than in HCs (0.96 ± 0.15 vs. 0.89 ± 0.14 mg/L, *p* = 0.024). The symbol ns is for *P* > 0.05, * for *P* < 0.05, ** for *P* < 0.01, and *** for *P* < 0.001, retrospectively.

Analysis did not identify a significant difference in the levels of Scr between the MSA patients and healthy subjects, as shown in Figure 2A (70.81 ± 13.88 vs. 70.92 ± 14.19 μmol/L, *p* = 0.967). However, the other three oxidative biomarkers were significantly higher in MSA patients than in healthy controls, as shown in Figures 2B–D (UA:325.31 ± 84.92 vs. 291.19 ± 64.14 μmol/L, *p* = 0.023; BUN:5.68 ± 1.67 vs. 4.60 ± 1.24 mmol/L, *p* < 0.001; Cys-C:0.96 ± 0.15 vs. 0.89 ± 0.14 mg/L, *p* = 0.024). Next, we compared these significantly elevated serum oxidative biomarkers between the two subgroups. Although we did not identify any significant differences, we found that the MSA-C patients exhibited relatively lower concentrations of these biomarkers than the MSA-P patients, as shown in Supplementary Figure 1 (UA:305.82 ± 72.89 vs. 348.04 ± 93.56, *p* = 0.074; BUN:5.44 ± 1.81 vs. 5.95 ± 1.49, *p* = 0.280; Cys-C:0.92 ± 0.14 vs. 1.00 ± 0.16, *p* = 0.093).

### Pearson Correlation Analysis

**FIGURE 3:**
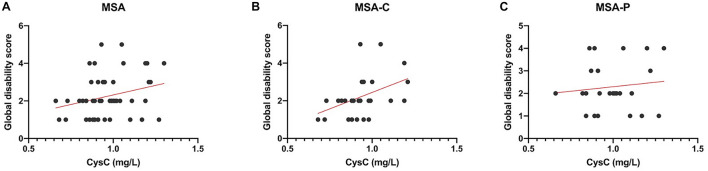
Pearson correlation analyses of serum Cys-C levels in MSA patients and subgroups. **(A)** The serum levels of Cys-C were positively correlated to GDS in MSA patients (*r* = 0.287, *p* = 0.039). **(B)** The serum levels of Cys-C were positively correlated to GDS in MSA-C patients (*r* = 0.444, *p* = 0.018). **(C)** The serum levels of Cys-C were not correlated to GDS in MSA-P patients (*r* = 0.118, *p* = 0.582).

Pearson correlation analyses revealed that there was no significant association between either UA and BUN with the UMSARS score, disease duration, or disease progression rate (Table 3). The serum levels of Cys-C were not associated with disease duration or disease progression rate but were significantly and positively correlated with GDS, as shown in Figure 3A (*r* = 0.287, *p* = 0.039), thus suggesting that this factor could act as a potential biomarker for patients with MSA. Next, we investigated the efficacy of serum Cys-C in the MSA-C and MSA-P subgroups. This serum biomarker showed a strong association with the MSA-C subgroup (Figure 3B; *r* = 0.444, *p* = 0.018) but not the MSA-P subgroup (Figure 3C; *r* = 0.118, *p* = 0.582). In addition, we explored the relationship between serum Cys-C and UMSARS scores in MSA-C patients. This serum biomarker was significantly positive correlated with disease duration (*r* = 0.407, *p* = 0.032) and GDS (*r* = 0.444, *p* = 0.018), but its relation to UMSARS-I (*r* = −0.115, *p* = 0.559), -II (*r* = 0.056, *p* = 0.779), total score (*r* = 0.042, *p* = 0.830) or the disease progression rates (*r* = −0.077, *p* = 0.696) was not statistical significance. These results suggest that serum Cys-C may act as a predictive factor for disease severity in patients with MSA-C.

**TABLE 3:** Pearson correlation analysis of the association between elevated biomarkers and disease severity in MSA patients.

	UA		BUN		Cys-C	
	*r*	*p*	*r*	*p*	*r*	*p*
UMSARS-I	−0.078	0.583	−0.041	0.774	−0.077	0.587
UMSARS-II	−0.089	0.530	0.009	0.952	−0.096	0.500
UMSARS-IV	0.084	0.554	0.028	0.844	0.287	0.039
Total score	−0.081	0.567	−0.011	0.939	−0.059	0.680
Disease duration	0.053	0.710	−0.097	0.493	0.048	0.736
Progression rate	−0.031	0.829	−0.063	0.656	−0.050	0.725

### The Predictive Value of Serum Cys-C in Patients With MSA-C

**FIGURE 4:**
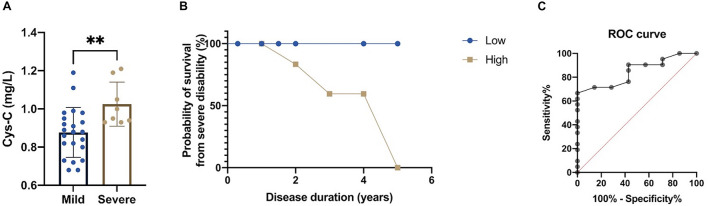
Evaluation of the predictive value of the serum levels of Cys-C in MSA-C patients. **(A)** A comparison of serum Cys-C levels between MSA-C patients with severe disability and those with mild disability (1.03 ± 0.13 vs. 0.88 ± 0.12 mg/L, *p* = 0.009). **(B)** Kaplan-Meier analysis of the 5-year probability of survival from MSA-C patients with severe disability when compared between those with high levels of Cys-C and those with low levels of Cys-C (Log-rank test: X^2^ = 4.154, *p* = 0.042). **(C)** ROC curve for serum Cys-C levels when predicting disease severity of patients with MSA-C (AUC = 0.847). The symbol ns is for *P* > 0.05, * for *P* < 0.05, ** for *P* < 0.01, and *** for *P* < 0.001, retrospectively.

In order to explore the predictive value of serum Cys-C for the disease severity of patients with MSA-C, we first classified disease severity into mild disability (GDS 1–2) and severe disability (GDS 3–5). We found that MSA-C patients with severe disability had significantly higher concentrations of serum Cys-C than patients with mild disability (1.03 ± 0.13 vs. 0.88 ± 0.12 mg/L, *p* = 0.009; Figure 4A), thus suggesting that high levels of serum Cys-C represents a positive risk factor for serious functional disability. Furthermore, MSA-C patients were divided into a high-concentration group and a low-concentration group based on mean serum Cys-C (0.92 mg/L) as a cut-off value. The 5-year probability of survival, based on severe disability, showed that MSA-C patients with high levels of Cys-C were associated with a significantly shorter duration of disease with severe functional disability, as determined by the Kaplan-Meier method (Figure 4B; Log-rank test: X^2^ = 4.154, *p* = 0.042). Finally, ROC curve analysis confirmed that this serum biomarker showed good performance as a predictor of disease severity in patients with MSA-C; the area under the curve (AUC) was 0.847 (Figure 4C).

## Discussion

Our analyses showed that there was no statistical difference between healthy controls and MSA patients with regards to serum Scr. However, the other three biomarkers were significantly higher in MSA patients than in controls. In addition, Pearson correlation analyses found that only serum levels of Cys-C were significantly correlated to GDS. Subgroup analysis also showed that serum Cys-C was positively associated with disease severity in patients with MSA-C; there was no association between serum Cys-C and disease severity in patients with MSA-P. Furthermore, MSA-C patients with severe disability had higher levels of serum Cys-C than those with mild disability. We also determined that the 5-year survival risk from severe disability was significantly increased in patients with MSA-C who also had high serum levels of Cys-C when compared to those with low levels of Cys-C.

Our analyses also indicated that there was a positive correlation between Cys-C concentration and disease severity in MSA patients; these findings were similar to a previous study involving other neurodegenerative disease (Hu et al., 2016). In addition, the increased Cys-C levels were found as an independent predictor for cognitive impairment via a meta-analysis including12 studies (Nair et al., 2020), and it could be used for distinguishing patients with cognitive impairment from healthy population (Wang et al., 2017). Shu et al. (2018) also revealed its predictive role in disease duration and severity in patients with anti-NMDAR encephalitis. These results suggested serum Cys-C might be a risk factor for neurological disorders. As a cysteine protease inhibitor enriched in the central nervous system, Suzuki et al. (2014) found that Cys-C was an oligodendrocyte-derived secretory protein led to MSA via overexpressing of human α-synuclein and via increased expression of the endogenous α-synuclein in a mouse model. Interestingly, Gauthier et al. (2011) reported that this serum factor played a protective role in brain damage and neurodegenerative processes by inhibiting cysteine proteases. Kaur et al. (2010) found that the over-expressed Cyst-C effectively rescued the neural injury by inhibiting the apoptosis-promoting actions of cathepsins in the mouse model of the inherited neurodegenerative disorder, progressive myoclonic epilepsy type 1 (EPM1). It’s worth noting that these animal models of neurodegenerative disorders were from the knock out of a single gene, while the susceptibility gene of MSA remained unclear. Simply, the homologous repair could be explained by the increased Cys-C levels in the EPM1 mouse model via knocking down Cystatin B. Thus, in our opinion, serum Cys-C may have multiple pathophysiological regulatory mechanisms in neurodegenerative disorders. When it comes to the relationship with MSA, serum Cys-C is more likely to be considered as a bad protein.

In a former study, Urbizu et al. (2015) confirmed that the transcription of Cys-C was differentially expressed in different MSA phenotypes. The Cys-C gene (CST3) B-haplotype was significantly associated with MSA-P, while the higher expression of CST3 were found in the caudate nuclei of MSA-P and in the cerebellum of MSA-C. Although our study did not prove a statistical difference in serum Cys-C between two phenotypes (only relatively lower expression in the MSA-C patients compared with MSA-P patients), a positive correlation between Cys-C and disease severity in MSA-C patients has been found out. Therefore, the serum levels of Cys-C may have a potential to evaluate the disease severity in patients with MSA-C while other variables should be taken into consideration together.

In contrast to the findings reported by Lee et al. (2011) and Cao et al. (2013), our study did not reveal an increase in the serum levels of Scr in MSA patients. Furthermore, we did not find an association between elevations of serum UA concentrations and the severity or progression of MSA. In our study, we did not explore the correlation between different quartiles of these serum oxidative biomarkers and MSA in order to avoid misclassification bias. In addition, we excluded patients with kidney disorders so that we could avoid individual bias; it is possible that our sample selection strategy may have contributed to the differences between our study and the findings of previous studies. However, Milanesi et al. (2019) found that UA was involved in the inflammatory reaction and oxidative stress mediated by the activation of toll-like receptor 4 (TLR4); this is the final oxidation product of purine catabolism in humans and primates and is known to exert neuroprotective effects. Zhang et al. (2017) revealed that higher UA concentrations could significantly improve cell viability and apoptosis, thus reducing the production of reactive oxygen species (ROS). These results might suggest that oxidative stress plays a key role in the mechanism underlying MSA.

A number of studies have shown that oxidative stress is a major factor in MSA. Zhou et al. (2016) found that increased serum levels of homocysteine and reduced serum levels of high-density lipoprotein cholesterol were potential prognostic biomarkers associated with the disease severity of MSA patients. In another study, Herrera-Vaquero et al. (2020) observed increased levels of tubulation in the mitochondria of neural progenitor cells from MSA patients and that ROS could promote the translocation of α-synuclein into nucleus, thus suggesting that oxidative stress plays a key role in MSA at early onset. In addition, Glat et al. (2020) found that oxidative stress-related genes were up-regulated in a mouse model of MSA and that an injection of mitochondrial neurotoxin could improve motor function. Collectively, these findings indicate that oxidative toxicity could represent a potential therapeutic target for MSA patients.

There are some limitations to our study that need to be considered. First, our study was limited by a small sample size of MSA patients. This is because we recruited patients from a single teaching hospital and some patients were reluctant to be involved in clinical studies. Therefore, further studies should now be carried out with a larger sample size and a longer follow-up period; such studies should provide us with a more comprehensive evaluation. Second, we only measured the concentrations of UA, Scr, BUN, and Cys-C once in this study. UMSARS assessments were conducted by independent neurologists. Therefore, random measurement errors may have influenced our data. Third, although MSA patients who took anti-PD drugs were excluded from this study, a few patients have previously taken oral mecobalamin (0.5 g/d). Future research should investigate the specific effect of mecobalamin on MSA patients.

## Conclusion

In conclusion, the serum levels of Cys-C may have a potential to evaluate the disease severity in patients with MSA-C while other variables should be taken into consideration together.

## Data Availability Statement

Original data for this study are included in the article/Supplementary Material, requests for further information can be obtained from the corresponding author.

## Ethics Statement

The studies involving human participants were reviewed and approved by the independent ethics committees of the First Affiliated Hospital of Chongqing Medical University and the First Affiliated Hospital of Sun Yat-sen University. The patients/participants provided their written informed consent to participate in this study.

## Author Contributions

FY designed the study, draft the manuscript, and contributed to the discussion. TW collected the data, analyzed the data, and contributed to the discussion. HL analyzed the data and draft the manuscript. JL and XW contributed to the discussion. WS designed the study and revised the manuscript. All authors contributed to the article and approved the submitted version.

## Conflict of Interest

The authors declare that the research was conducted in the absence of any commercial or financial relationships that could be construed as a potential conflict of interest.

## Publisher’s Note

All claims expressed in this article are solely those of the authors and do not necessarily represent those of their affiliated organizations, or those of the publisher, the editors and the reviewers. Any product that may be evaluated in this article, or claim that may be made by its manufacturer, is not guaranteed or endorsed by the publisher.

## Supplementary Material

Supplementary Figure 1 Serum oxidative biomarkers in different MSA subgroups. **(A)** There was no significant difference in the serum levels of UA when compared between patients with MSA-C and MSA-P (305.82 ± 72.89 vs. 348.04 ± 93.56, *p* = 0.074). **(B)** There was no significant difference in the serum levels of BUN when compared between patients with MSA-C and MSA-P (5.44 ± 1.81 vs. 5.95 ± 1.49, *p* = 0.280). **(C)** There was no significant difference in the serum levels of Cys-C when compared between patients with MSA-C and MSA-P (0.92 ± 0.14 vs. 1.00 ± 0.16, *p* = 0.093).
