# Supplementary figures and images for: Serum Cystatin C as a Potential Predictor of the Severity of Multiple System Atrophy With Predominant Cerebellar Ataxia: A Case-Control Study in Chinese Population
Source: Front Neurosci. 2021 Sep 10;15:663980. doi: 10.3389/fnins.2021.663980 (PMC8461053; doi:10.3389/fnins.2021.663980)

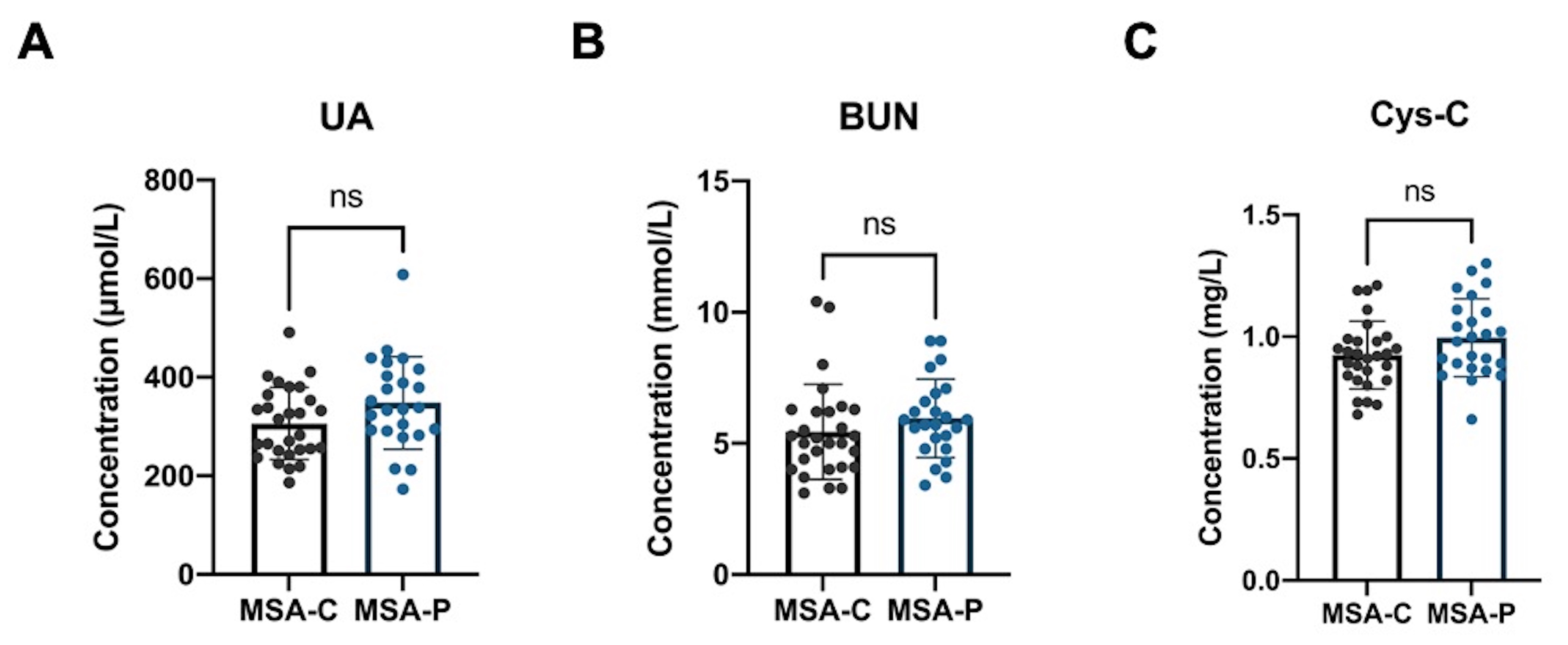

Supplement: Supplementary Figure 1 — Serum oxidative biomarkers in different MSA subgroups. (A) There was no significant difference in the serum levels of UA when compared between patients with MSA-C and MSA-P (305.82 ± 72.89 vs. 348.04 ± 93.56, p = 0.074). (B) There was no significant difference in the serum levels of BUN when compared between patients with MSA-C and MSA-P (5.44 ± 1.81 vs. 5.95 ± 1.49, p = 0.280). (C) There was no significant difference in the serum levels of Cys-C when compared between patients with MSA-C and MSA-P (0.92 ± 0.14 vs. 1.00 ± 0.16, p = 0.093). [file Image_1.JPEG]
